# Effects and safety of endovascular recanalization for non-acute symptomatic intracranial vertebral artery occlusion with different risks

**DOI:** 10.1097/MD.0000000000036813

**Published:** 2024-02-16

**Authors:** Shunqiang Chen, Jinchao Xia, Shuxin Xiao, Tianxiao Li, Ziliang Wang

**Affiliations:** a Henan Provincial Intervention Center, Zhengzhou University People’s Hospital, Henan Provincial People’s Hospital, Zhengzhou, Henan, China; b Department of Cerebrovascular Disease, Zhengzhou University People’s Hospital, Henan Provincial People’s Hospital, Zhengzhou, Henan, China.

**Keywords:** endovascular recanalization, intracranial, non-acute phase, symptomatic, vertebral artery

## Abstract

There is no consensus on the optimal treatment for non-acute symptomatic intracranial vertebral artery occlusion, and endovascular recanalization is a challenging procedure. We report our clinical experience of endovascular recanalization in patients with non-acute symptomatic intracranial vertebral artery occlusion to assess the feasibility and safety of endovascular recanalization and determine the candidate patients for this procedure. Ninety-two patients with non-acute symptomatic intracranial vertebral artery occlusion who underwent endovascular recanalization from January 2019 to December 2021 were retrospectively analyzed. we grouped all patients according to imaging examination findings, occlusion length, duration, nature, calcification, and angulation to evaluate the risk of endovascular recanalization. The overall success rate of endovascular recanalization was 83.7% (77/92), and the perioperative complication rate was 10.9% (10/92). Among the 3 classification groups, the recanalization success rate gradually decreased from the low-risk group to the high-risk group (low-risk: 100%, medium-risk: 93.3%, high-risk group: 27.8%, *P* = .047), while the overall perioperative complication rate showed the opposite trend (0%, 10.0%, 38.9%, respectively, *P* = .001); the proportion of patients with 90-day modified Rankin Scale scores of 0–2 decreased successively (100%, 83.3%, and 22.2%, respectively, *P* < .026); 77 patients with successful recanalization were followed; the rate of restenosis/reocclusion increased sequentially (0%, 17.9%, and 80%, respectively, *P* = .000). Patients in the low- and medium-risk groups showed a good clinical course after endovascular recanalization. Among 88 patients (four patients lost to follow-up), with a median clinical follow-up of 13 months (interquartile range ¼, 7–16), the rate of stroke or death after 30 days was 17.4% (16/92). Endovascular recanalization is safe and feasible for low- and medium-risk patients with non-acute symptomatic intracranial vertebral artery occlusion; it is also an alternative to conservative therapy for the patients.

## 1. Introduction

Acute intracranial vertebral artery (ICVA) occlusion is associated with high mortality and morbidity, current randomized controlled trials and meta-analyses of observational studies recommend endovascular recanalization for acute vertebral artery occlusion.^[1]^ However, some patients with ICVA occlusion survive the acute stage of arterial occlusion and enter non-acute stage. Although these patients survive the acute stage and receive aggressive clinical management, Some patients with hemodynamic damage or extension of the occlusive lesions present with progressive or recurrent symptoms.^[2,3]^ Some patients experience recurrent symptoms; repeated infarctions of the brain stem and cerebellum can lead to catastrophic results, especially in those with hemodynamic instability or occlusive progressions.^[4–6]^ There is no consensus on the optimal management of patients with non-acute ICVA occlusion. A surgical bypass procedure for this condition has been described previously; however, the bypass procedure is technically challenging and confers a greater risk for complications than anterior circulation.^[7,8]^ A few small case series suggest that endovascular recanalization may be a viable treatment option for patients with non-acute symptomatic ICVA occlusion and that the success rate of endovascular recanalization and perioperative complications are influenced by occlusion length, duration, nature, calcification, angulation, and etiology.^[6,9–12]^

In this study, we report our single-center clinical experience with endovascular recanalization in patients with non-acute symptomatic ICVA occlusion to assess its feasibility and safety. In addition, we grouped all patients according to imaging examination findings and occlusion length, duration, nature, calcification, and angulation to evaluate the risk of endovascular recanalization. The patients were scored and classified to evaluate the technical risk and perioperative complications of endovascular recanalization surgery, explore the feasibility and safety of endovascular recanalization for non-acute symptomatic ICVA occlusion, and provide a reference for patient selection and risk stratification.

## 2. Materials and methods

**Figure 1.**
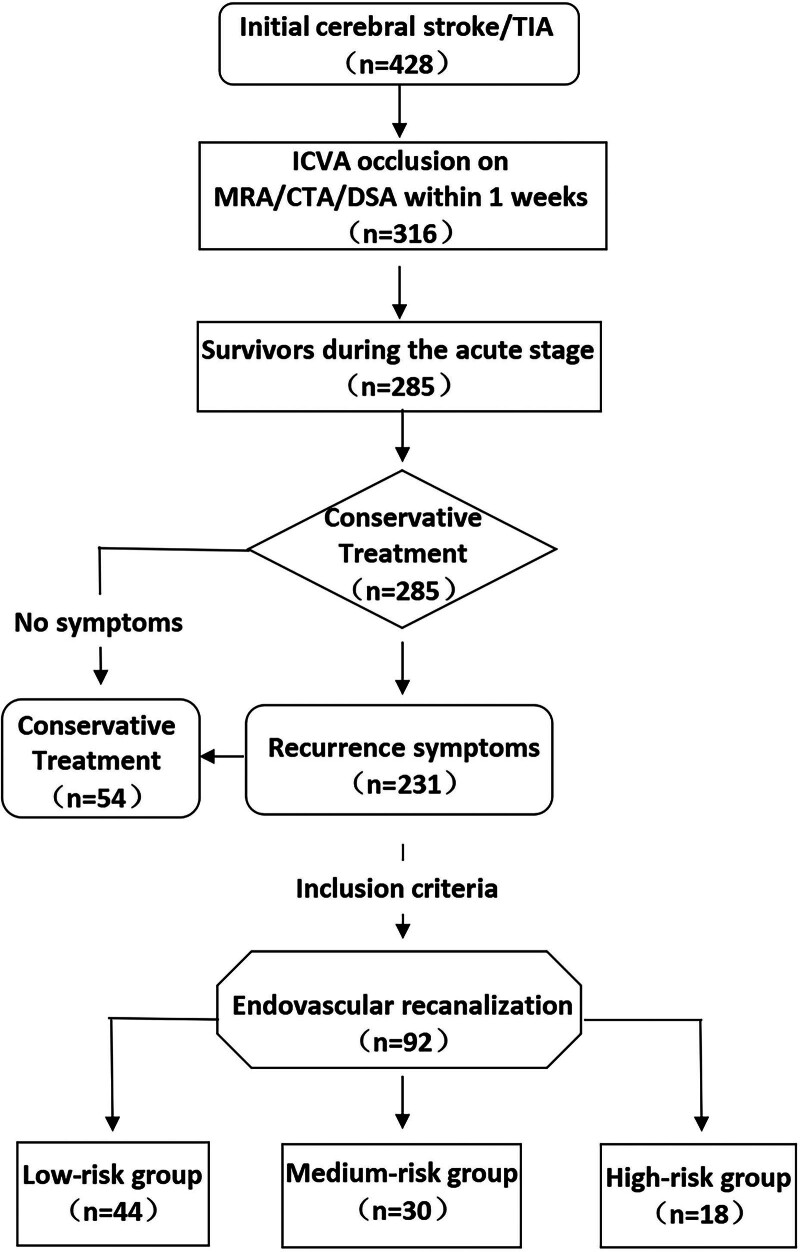
Flow chart of patient enrollment. CTA = computed tomography angiography, DSA = digital subtraction cerebral angiography, MRA = magnetic resonance angiography, TIA = transient ischemic attack.

From January 2019 to December 2021, 92 patients with endovascular recanalization for non-acute symptomatic ICVA occlusion were retrospectively analyzed at the stroke center of our hospital. The flow chart of enrollment of patients is shown in Figure 1. Our sample included 26 females and 66 males, with an average age of 58.0 ± 8.9 years (range, 39–80 years); patients with ICVA occlusion were mainly diagnosed using magnetic resonance angiography (MRA) or computed tomography angiography (CTA), which was performed to confirm the diagnosis when patients showed clinical symptoms (stroke or transient ischemic attack [TIA] within 24 hours after onset); the patients received dual antiplatelet therapy, along with intensive management of risk factors (dyslipidemia, abnormal blood sugar metabolism, blood pressure, obesity, smoking, physical inactivity, etc) and/or lifestyle changes, but the clinical symptoms still recurred.^[13]^ Intensive management of risk factors was defined as low-density lipoprotein (LDL), namely atorvastatin (40–80 mg/day) or rosuvastatin (20–40 mg/day), and the statin dose was adjusted to achieve or reduce low-density lipoprotein levels below 70 mg/dL. Symptomatic disease was defined as the presence of progressive or recurrent symptoms of vertebrobasilar ischemia confirmed clinically and radiographically. An increase in the National Institutes of Health Stroke Scale (NIHSS) score of ≥ 4 points at 72 hours after symptom onset indicated the presence of progressive symptoms.^[10]^ Recurrent TIA was defined as acute episodes of vertebrobasilar ischemic symptoms that resolved within 24 hours or persistent worsening of vertebrobasilar insufficiency in an upright posture that resolved with postural correction. Recurrent stroke was defined as any new focal neurologic deficit associated with non-acute ICVA occlusion lasting more than 24 hours and confirmed clinically and radiographically. The study protocol was approved by the Institutional Review Board of Zhengzhou University People Hospital (Henan Provincial People Hospital). All patients gave informed consent and their anonymity was preserved.

The inclusion criteria were as follows: presence of non-acute symptomatic ICVA occlusion diagnosed by MRA or CTA and confirmed by digital subtraction angiography (DSA); presence of progressive and recurrent stroke or TIA despite optimal medical treatment; presence of a small infarct core, with a posterior circulation Alberta Stroke Program Early CT Score of ≥ 6 points^[14]^; presence of neurological deficits (NIHSS) that did not match the MRI diffusion-weighted imaging infarct core (clinical-image mismatch), determined by a 3-person expert panel consultation and based on the majority opinion of the physician judgment^[10]^; and presence of at least 1 risk factor for atherosclerosis (such as age ≥ 60 years, current smoker status, hypertension, hyperlipidemia, or diabetes).

The exclusion criteria were as follows: patients without risk factors for atherosclerosis identified as having arterial dissection, Moya-Moya syndrome, or arteritis based on clinical symptoms, imaging data, and/or tests; patients with cardiac cerebral embolism (such as atrial fibrillation, bacterial endocarditis, mitral stenosis, myocardial infarction within 6 weeks, prosthetic valve, and ventricular aneurysm); patients with intracranial aneurysm or hemorrhagic disease; and patients with life expectancy < 1 year due to other health problems were excluded.

Patients with non-acute symptomatic ICVA occlusion were divided into 3 groups according to the imaging data and occlusion length, duration, nature, calcification, and angulation (Table 1): low-risk group: 0 to 2 points; medium-risk group: 3 to 5 points; high-risk group: 6 to 8 points. The occlusion length was defined as the distance between the proximal end of the occlusion and the visible distal segment on preoperative imaging (CTA or DSA). Occlusion duration was defined as 100% occlusion of the vascular lumen cross-section on CTA or MRA, with occlusion confirmed by DSA and the duration from the date of initial diagnosis to the day of surgery determined by the majority opinion of a 3-person expert panel consultation. Each patient history of atherosclerosis risk factors and preoperative examination data were used to confirm the diagnosis. Occlusion calcification was defined as “any form of calcified lesion within the outline of the blood vessel” on CT, determined by the majority opinion of a 3-person expert panel consultation. Occlusion angulation was defined as the angle of the intersection between the distal and proximal segments of the occluded line. In some cases, there were 2 or more occluded segments, and the maximum angle was used as the occlusion angulation.

**Table 1:** Scores for endovascular recanalization grouping of non-acute symptomatic ICVA occlusion.

Variables	Score
Length of occlusion	
≤5 mm	0
5–10 mm	1
≥10 mm	2
Occlusion duration	
≤1 m	0
1–3 m	1
>3 m	2
Occlusion calcified	
No	0
Yes	1
Occlusion nature	
Atherosclerotic	0
Others*	1
Occlusion angulation	
Straight	0
Angulation ≤ 30°	1
Angulation > 30°	2
Total score:	
0–2	Low risk
3–5	Medium risk
6–8	High risk

Note: ICVA, intracranial vertebral artery; Other*, occlusion due to inflammation, dissection, radiation, or fibromuscular dysplasia.

All surgical procedures were performed by experienced neurointerventional doctors under general anesthesia. The 6F femoral artery sheath was implanted, and heparin was administered to achieve an activated clotting time of 200 to 300s. For patients with compensated circulation through the posterior communicating artery (PCOM) in the distal segment of the occlusion, the dual roadmap technique with simultaneous injection of vertebral and internal carotid arteries was used to guide the catheter in the axial direction of the occlusion site.^[15]^ A microcatheter (Excelsior SL-10, Stryker, California, USA or Echelon-10, EV3, California, USA) combined with a micro-guide wire (Transend EX 0.014/205 soft tip, Boston Scientific Corporation, Massachusetts, USA or Synchro-200cm, Stryker, California, USA) were carefully passed through the occlusion segment. If the microcatheter and micro-guide wire could not pass through the distal true lumen after attempts, the procedure was terminated. Once the microcatheter entered the real lumen, it was placed at the distal end of the occlusion, and a 300cm long micro-guide wire (Transend EX 0.014, Boston Scientific Corporation, Massachusetts, USA or Synchro, Stryker, California, USA) was used to exchange the microcatheter along the exchange micro-guide. A 1.5mm–3mm balloon (Gateway, Stryker, USA; Sprinter, Medtronic, USA; or Sino, Sino Medical, China) was placed along the microwire and inflated to 6atm for 60seconds. Based on the operator experience, balloon-expandable or self-expanding stents (Apollo, MicroPort, China; Resolute, Medtronic, USA; Neuroform EZ, Stryker, USA; LVIS, Microvention Company, USA; or Wingspan, Stryker, USA) were deployed. Postoperative angiography was performed to confirm the blood flow of the vertebral artery, and a modified thrombolysis in cerebral infarction (mTICI) grade of ≥ 2b and residual stenosis < 50% were defined as successful recanalization. A brain CT was immediately performed to determine whether there was any intracranial hemorrhage after the surgery. If the patient had aggravated or new stroke symptoms after treatment, brain CT or MRI was required to confirm the diagnosis of stroke. After surgery, the systolic blood pressure of the patients was controlled at ≤ 120 mm Hg to reduce the risk of reperfusion hemorrhage.

The patients took both clopidogrel (75 mg) and aspirin (100 mg) for at least 5 days before the surgery; those who received dual antiplatelet therapy for <5 days should be given Tirofiban after surgery and platelet reactivity should be evaluated by thromboelastography before the procedure.^[16]^ Aspirin resistance was defined as arachidonic acid-induced platelet aggregation inhibition < 50%, and clopidogrel resistance was defined as < 30% inhibition of ADP-induced platelet aggregation. None of the patients in this group had aspirin resistance; 14 patients were resistant to clopidogrel, so ticagrelor (90 mg) was used twice daily as a replacement for clopidogrel. Postoperative dual antiplatelet were continued for 6 months, followed by life-long single antiplatelet therapy (oral clopidogrel 75 mg or aspirin 100 mg).

Follow-up clinical and imaging examinations were performed after surgery, and the clinical, imaging, and laboratory test data of the patients were collected. All imaging assessments were performed by 2 independent neurointerventional specialists, and all differences were resolved by consensus. In-stent restenosis was defined as angiographically proven stenosis on either side of the stent within 5 mm of the stent margin, diameter stenosis of > 50%, and lumen diameter loss of 20% immediately after surgery.^[17]^ The modified Rankin Scale (mRS) scores were divided into good (0–2 points), moderate (3 points), and poor (4–6 points).

## 3. Statistical analysis

All statistical tests were performed using the SPSS version 20.0. Normally distributed quantitative variables are presented as mean ± standard deviation for all continuous variables, non-normally distributed variables are presented as median and interquartile range, and categorical variables are presented as numbers and proportions. Statistical differences between 2 groups were assessed using Student *t* test or approximate t-test if the variance between the groups was significantly different. The differences between 3 groups were evaluated using the χ^2^ test for trends. Differences were considered to be statistically significant at *P*<.05.

## 4. Results

All 92 patients underwent endovascular recanalization for nonacute symptomatic ICVA occlusion. The median time from the last symptom onset to endovascular treatment was 27 days (IQR, 20–41.25 days). These patients were divided into 3 groups, low-risk(44 patients), medium-risk (30 patients), and high-risk (18 patients), according to their preoperative data. There were no significant differences in the demographic characteristics, risk factors, or clinical characteristics of the patients among the 3 groups. The detailed baseline characteristics of the patients are summarized in Table 2.

**Table 2:** Baseline characteristics of 92 patients with non-acute symptomatic ICVA occlusion.

Variables	Total (n = 92)	Low-risk group (n = 44)	Medium-risk group (n = 30)	High-risk group (n = 18)	χ^2^/F	*P*
F/M	26/66	13/31	8/22	5/13	0.042	.979
Age (yr), mean ± SD	58.0 ± 8.9	59.4 ± 9.6	57.4 ± 7.6	55.4 ± 8.9	1.386	.255
Height (cm), mean ± SD	168.0 ± 6.9	167.3 ± 7.5	168.2 ± 5.9	169.4 ± 7.0	0.604	.549
Weight (kg), mean ± SD	73.0 ± 9.3	72.9 ± 9.0	73.3 ± 9.2	72.9 ± 10.6	0.014	.986
BMI, mean ± SD	26.1 ± 3.3	26.5 ± 3.7	25.9 ± 2.8	25.4 ± 3.3	0.739	.480
Infarction/TIA(n)	85/7	40/4	29/1	16/2	0.048	.976
Hypertension, n	63	37	20	11	0.711	.701
Diabetes mellitus, n	32	15	13	4	1.112	.573
Hyperlipidemia, n	29	14	11	4	0.589	.745
Alcohol drinking, n	38	15	12	11	1.483	.476
Smoking, n (%)	43	18	16	9	0.436	.804
Pre-surgery mRS n:					0.121	.886
0–2	92	44	30	18		
3–6	0	0	0	0		

BMI = body mass index, F/M = female/male, ICVA = intracranial vertebral artery, mRS = modified Rankin scale, TIA = transient ischemic attack.

**Figure 2.**
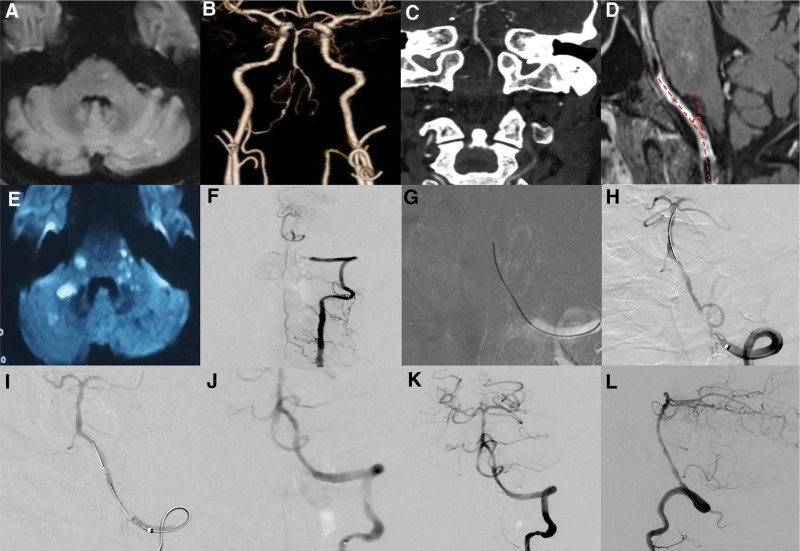
A 66-yr-old male patient with non-acute symptomatic ICVA occlusion in the low-risk group, chief complaint: paroxysmal dizziness for 1 mo, aggravated with slurred speech for 13 days; past medical history: hypertension for 6 yr; physical examination: slurred speech, the limb muscle strength and tone was normal; the mRS score was 1. (A) Preoperative magnetic resonance imaging (MRI) shows acute ischemic stroke in the right lateral brainstem and cerebellum. (B–C) Computed tomography angiography (CTA) shows that the left vertebral artery is dominant, and the left intracranial vertebral artery is occluded, and no calcification is found in the occluded segment. (D) High-resolution MRI shows that the occlusion length is <10 mm, the occlusion angulation was <30°. (E) MRI shows new acute bilateral pontine infarction after symptoms aggravated. (F) Preoperative digital subtraction angiography (DSA) shows that the left intracranial vertebral artery was occluded, and the occlusion distal end was compensated by the anterior spinal artery. (G–I) Microcatheter (SL-10) and micro-guide wire (Synchro) passed through the occlusion segment, dilated with Gateway balloon (2.0 mm × 15.0 mm), and deployed Enterprise (4.5 mm × 22.0 mm) Stent. (J) DSA shows successful recanalization, cTIMI grade: III. (K–L) 6 mo follow-up DSA shows smooth blood flow in the stent and distal vessels. The patient was followed up for 13 mo, no recurrence stroke, and mRS score was 0. ICVA = acute intracranial vertebral artery, mRS = modified Rankin Scale scores.

The overall technical success rate was 83.7% (77/92), perioperative complication rate was 10.9% (10/92), and overall rate of stroke or death within 30 days was 5.4% (5/92). Among the 3 classification groups, the recanalization success rate gradually decreased from the low-risk group to the high-risk group (100%, 93.3%, and 27.8%, respectively; *P* = .047), while the overall perioperative complication rate showed the opposite trend (0%, 10.0%, and 38.9%, respectively; *P* = .001); the proportion of 90-day mRS score of 0–2 decreased successively (100%, 83.3%, and 22.2%, respectively; *P* < .026); 77 patients with successful recanalization were followed, and the rate of restenosis/reocclusion increased sequentially (0%, 17.9%, and 80%, respectively, *P* = .000). Table 3 lists the detailed surgical success rates, perioperative complications, and follow-up results. Among the 77 patients with successful recanalization, 1 patient suffered hyperperfusion cerebral hemorrhage after successful perioperative recanalization, and the symptoms had completely resolved after 12 days of treatment. Another patient developed a new neurologic symptom (TIA attack) after recanalization, and the symptoms had completely resolved after treatment; cerebral infarction occurred in 2 patients due to embolism during surgery. The operation was terminated in 15 patients because the micro-guide wire could not pass through the occlusion segment to the true lumen of the distal vessel. Among them, 4 patients experienced ischemic stroke (NIHSS score increase ≥ 4 points), and 2 experienced intracranial hemorrhage during the perioperative period. The median clinical follow-up period of the 88 patients (4 patients were lost to follow-up) was 13 months (IQR ¼, 7–16), the rate of stroke or mortality beyond 30 days was 17.4% (16/92); there were 2 deaths (due to either cerebral hemorrhage or ischemic stroke), 7 patients experienced major ischemic stroke (NIHSS score > 15, mRS, 4–5), and 7 patients had mild ischemic stroke (NIHSS score ≤ 4). The median imaging follow-up duration was 4 months (IQR ¼, 3–7) for 89 patients (three were lost), in-stent reocclusion occurred in 3 of 77 patients with successful recanalization; all 3 patients had ipsilateral ischemic stroke, and DSA/CTA showed in-stent reocclusion (no further surgery was performed); their symptoms improved after medication and rehabilitation. Six patients developed in-stent restenosis (two patients experienced ipsilateral ischemic stroke and 2 patients had no new symptoms). Although the 12 patients with recanalization failure (three were lost to follow-up) received drug treatment, 2 died and the remaining patients all had new ischemic stroke (NIHSS score ≥ 4 points) (Fig. 2 shows the operative and follow-up data of a patient with non-acute symptomatic ICVA occlusion in the low-risk group).

**Table 3:** Complications and clinical outcomes of 92 patients with non-acute symptomatic ICVA occlusion.

Variables	Total (n = 92)	Low-risk group (n = 44)	Medium-risk group (n = 30)	High-risk group (n = 18)	χ^2^/F	*P*
Recanalization rate, n (%)	77 (83.7%)	44 (100%)	28 (93.3%)	5 (27.8%)	6.134	.047
Treatment methods, n					1.801	.172
BD only	13	9	3	1		
BD + SE stent	47	32	14	1		
BD + BE stent	8	2	5	1		
BD + BE + SE stent	9	1	6	2		
mTICI grade(n)					0.234	.885
IIb	36	19	14	3		
III	41	25	14	2		
Complication rate, n (%)	10 (10.9%)	0 (0)	3 (10.0%)	7 (38.9%)	14.162	.001
Hemorrhagic complications	3	0	1	2		
Ischemic complications	7	0	2	5		
Stroke within 30 d, n(%)	5 (5.4%)	0 (0)	1 (3.3%)	4 (22.2%)	10.264	.006
90-d mRS n					7.272	.026
0–2	73	44	25	4		
3–6	15	0	4	12		
Stroke and death beyond 30 d, n	16	0	4	12	22.981	.000
Restenosis, n (%)	6 (7.8%)	0 (0)	4 (14.3%)	2 (40.0%)	9.509	.009
Reocclusion, n (%)	3 (3.9%)	0 (0)	1 (3.6%)	2 (40.0%)	13.670	.001

BE = ball-expandable stent, BD = balloon dilatation, ICVA = intracranial vertebral artery, mRS = modified Rankin scale, mTICI = modified thrombolysis in cerebral infarction, SE = self-expandable stent.

## 5. Discussion

In this study, we report our single-center clinical experience with endovascular recanalization in patients with non-acute symptomatic ICVA occlusion. The results showed that endovascular recanalization of non-acute symptomatic ICVA occlusion is feasible, and the overall recanalization rate in this case series was 83.7%. The success rate decreased from the low-risk group to the high-risk group, whereas the incidence of perioperative complications increased. Therefore, non-acute symptomatic ICVA occlusion may lead to different recanalization outcomes, depending on the type of lesion causing it. Our results are consistent with most recent case-series studies which reported that successful recanalization of nonacute symptomatic ICVA occlusion has been reported to range 70% to 90% with a periprocedural complication rate of 10%-20%.^[4,6,9,18]^

The optimal timing of endovascular recanalization for non-acute symptomatic ICVA occlusion remains unclear. Previous studies on the treatment of non-acute coronary artery total occlusion have shown that the successful recanalization rate is higher when the occlusion length is shorter and the occlusion duration is <3 months; however, severe calcification and extreme angulation of the occluded segment increase the risk of recanalization failure.^[19–21]^ Studies on non-acute intracranial artery occlusion have also shown a high success rate when the duration is < 3 months.^[20]^ In our case series, the median time from last symptom onset to endovascular treatment was 27 days (IQR, 20–41.25 days); however, it should be noted that early recanalization in patients with ischemic stroke may result in reperfusion hemorrhage or hemorrhagic transformation since the capillary bed in patients with acute stroke is weakened.^[22]^

When the intracranial vertebral artery is occluded, the PCOM in the anterior circulation or meningeal branch of the cerebellar arteries can compensate for the occluded distal vascular collaterals. For patients with non-acute symptomatic ICVA occlusion, if a visual reconstruction of collaterals distal to the occlusion is performed, a dual or long-term roadmap mode can be used during endovascular therapy to visualize both distal and proximal vessels, roughly define the occlusion segment, and guide the microcatheter/microwire to the required route.^[15]^ A history of ICVA occlusion may be closely related to collateral compensation, and recurrent or progressive ischemic symptoms suggest hemodynamic impairment or the presence of progressive occlusive lesions, which need to be actively treated with surgery. In this case series, the lesions of patients in the low-risk and intermediate-risk groups showed good recanalization results and they could be the most suitable candidates for endovascular recanalization; for the patients in the high-risk group, the recanalization rate was low (27.8%) and the rate of perioperative complications was high (38.9%); therefore, endovascular recanalization should be performed cautiously for high-risk patients. In addition, it is important to note that endovascular recanalization for non-acute symptomatic ICVA occlusion is still a high-risk procedure and should be performed by a skilled operator.

Perioperative complications of endovascular recanalization for non-acute symptomatic ICVA occlusion include ischemic complications (such as stroke, vascular dissection, acute thrombosis, and distal embolization) and hemorrhagic complications (such as perforation of occluded vessels and reperfusion hemorrhage).^[23,24]^ In this case series, the incidence of perioperative complications was 10.9% (10/92). Among the 77 patients with successful recanalization, 3 suffered ischemic complications (one patient had new neurological symptoms [TIA attack]) that had completely resolved after treatment; cerebral infarction occurred in 2 patients due to embolism during surgery) and 1 developed hemorrhagic complications (the patient suffered hyperperfusion cerebral hemorrhage after successful recanalization, and after 12 days of treatment, the symptoms had completely resolved). Among the 15 patients with failed recanalization, ischemic stroke (NIHSS score ≥ 4) occurred in 4, and postoperative intracranial hemorrhage occurred in 2 patients. Previous studies have shown that perioperative complications of angioplasty for intracranial stenosis are associated with a high risk of ischemic stroke.^[25,26]^ In the SAMMPRIS trial, the proportion of patients who experienced ischemic stroke caused by perforating artery occlusion within 1 month after intracranial angioplasty was relatively high, especially in the branches enriching the responsible vessel, which may be due to the displacement, destruction, or snow-plowing of atherosclerotic plaques.^[22]^ Mori et al also classified the levels of intracranial atherosclerotic stenosis and found that lesion length correlated with perioperative complications and technical success rates.^[27]^ Therefore, in patients with occlusions, displacement of atherosclerotic plaques, progressive local thrombus, and migration of blood clots may increase the risk of perforator occlusion. Thrombosis caused by suspending antiplatelet drugs after hemorrhagic complications is also an important factor for recanalization of non-acute symptomatic ICVA occlusions.^[28]^

This study has some limitations. First, the study was a single-center study with small sample size, lack of quantitative perfusion studies comparing regional blood volumes, Chinese patients enrolled only and on-randomized controlled trials, which may all produce some bias to affect the generalization of the study. Second, our study did not include a control group receiving medical management; a few randomized trials of endovascular versus medical therapy for intracranial stenosis have shown a lower risk with medical therapy.

## 6. Conclusion

Endovascular recanalization is likely to be a salvage treatment option for patients with non-acute symptomatic ICVA occlusion; therefore, identifying the most appropriate patients for endovascular recanalization is strongly recommended. Endovascular recanalization is safe and feasible in reasonably selected patients with non-acute symptomatic ICVA occlusion (particularly in the low- and medium-risk groups). Furthermore, grouping of patients based on occlusion length, duration, nature, calcification, and angulation can help clinicians identify suitable candidates for endovascular recanalization.

## Author contributions

**Conceptualization:** Tianxiao Li.

**Formal analysis:** Shuxin Xiao.

**Project administration:** Shunqiang Chen.

**Writing – original draft:** Ziliang Wang.

**Writing – review & editing:** Jinchao Xia.
